# Acenocoumarol’s pharmacokinetic: linear or not?

**DOI:** 10.17179/excli2019-1714

**Published:** 2019-08-27

**Authors:** Parveen Kumar, Puneet Kapoor, Chhikara Meenu

**Affiliations:** 1 Pharmalex India Pvt Ltd., New Delhi, India; 2 School of Pharmaceutical Education and Research, Jamia Hamdard, New Delhi, India

## ⁯⁯

***Dear Editor,***

Acenocoumarol, is a racemic mixture of the optical R (+) and S (-) enantiomers. R (+) enantiomer is several times more potent than the S (-) enantiomer (Godbillon et al., 1981[1]). Acenocoumarol is rapidly absorbed following oral absorption with approximately 60 % of the dose available systemically (Trailokya, 2015[13]). After a single dose of 10 mg, the peak plasma concentrations (C_max_) of acenocoumarol are reached within 1-3 h and the area under the plasma concentration-time curve (AUC) values are proportional to the dose in the dosage range of 8 to 16 mg (Sasso et al., 2012[8]). The protein binding of acenocoumarol is 98 % (Trailokya et al., 2016[14]). Acenocoumarol is mainly metabolized by CYP2C9 (Trailokya, 2015[13]); 6- and 7-hydroxylation of both enantiomers of acenocoumarol are the major metabolites (Thijssen et al., 2000[11]). The elimination half-life of acenocoumarol is 8 to 11 h (Sánchez et al., 2013[7]). Approximately, 29 % of acenocoumarol excrete in feces and 60 % in urine. The starting dose of acenocoumarol usually ranged from 2 to 4 mg. Based on the prothrombin time, subsequent loading doses may be recommended (Trailokya, 2015[13]).

Acenocoumarol is reported to exhibit a dose-proportional pharmacokinetics for the 8 to 16 mg doses (Trailokya, 2015[13]). However, no information is available for the dose-proportionality of lower doses of acenocoumarol (i.e. 1 to 4 mg doses). We aimed to evaluate the dose-proportionality of acenocoumarol by performing a literature search and plotting a linear curve for AUC vs. dose from the available information.

**Figure 1:**
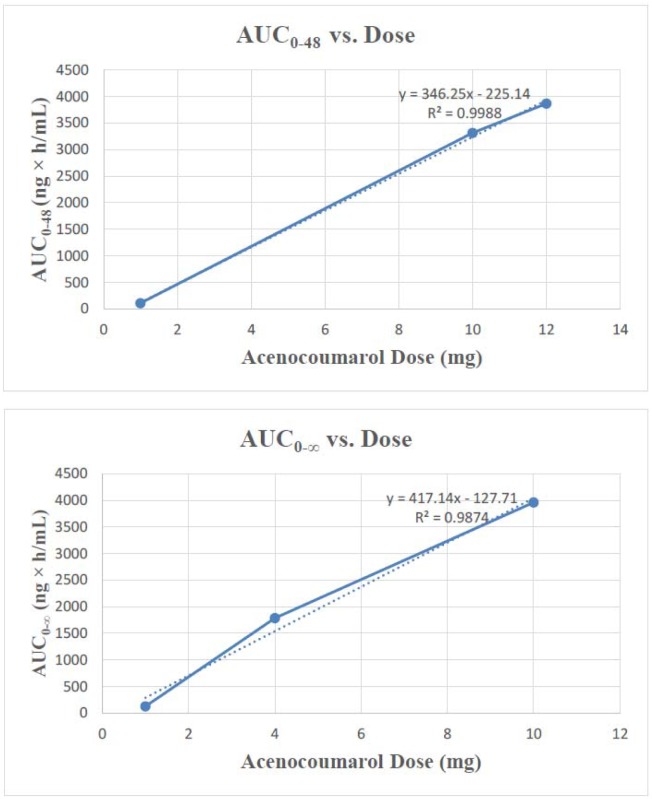
Dose-proportionality curves between AUC and acenocoumarol doses (AUC_0-48 _vs. dose, and AUC_0-∞_ vs. dose)

Literature related to pharmacokinetics of acenocoumarol was searched in PubMed. A total of 115 from 1618 articles were identified related to acenocoumarol's pharmacokinetics. From, 115 articles, 9 articles were identified as potentially relevant, as these articles reported the AUC values at different time points such as 24, 48, 72 h and at infinite time. These articles were finally considered for the evaluation of linearity of acenocoumarol pharmacokinetics. Various studies have reported the AUC_0-48_ and AUC_0-∞_ values of acenocoumarol for 1, 4, 10 and 12 mg dose (Table 1(Tab. 1); References in Table 1: Huang et al., 2008[2]; Masche et al., 1999[3]; Popovic et al., 1994[4]; Rolan et al., 2003[6]; Sasso et al., 2012[8]; Sunkara et al., 2004[9]; Thijssen and Baars, 1983[10]; Thijssen and Hamulyàk, 1989[12]). No other information on AUC_0-48_ and AUC_0-∞_ were available with the 2, 8 and 16 mg dose. The pharmacokinetics data across these studies were used to generate a dose-proportionality curve (acenocoumarol dose vs. AUC_0-48_ or acenocoumarol dose vs. AUC_0-∞_). The dose-proportionality curves between AUC and acenocoumarol doses (AUC_0-48 _vs. dose, and AUC_0-∞_ vs. dose) are presented in Figure 1(Fig. 1). 

An R^2^ of 1 indicates that the regression predictions perfectly fit the data. Therefore, from the value of R^2^ (0.9988 for AUC_0-48 _vs. dose, and 0.9874 for AUC_0-∞ _vs. dose), it is clear that acenocoumarol exhibits a dose-proportional pharmacokinetics.

## Figures and Tables

**Table 1:**
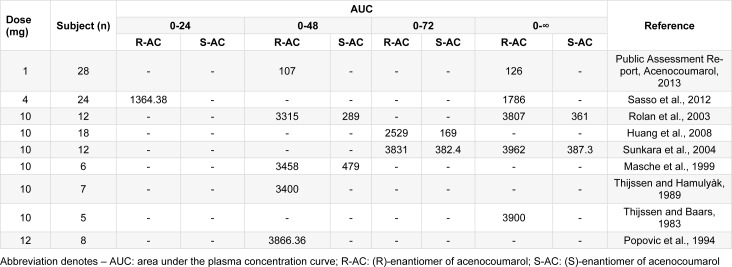
AUC_0-48_ and AUC_0-∞_ values of acenocoumarol from literature search
